# Herpes Simplex Viral Encephalitis Masquerading as a Classic Left MCA Stroke

**DOI:** 10.1155/2015/673724

**Published:** 2015-12-03

**Authors:** Peter A. Abdelmalik, Timothy Ambrose, Rodney Bell

**Affiliations:** ^1^Department of Anesthesia and Critical Care Medicine, Neurosciences Division, Johns Hopkins University School of Medicine, Baltimore, MD 21287, USA; ^2^Department of Neurology, Thomas Jefferson University Hospitals, Philadelphia, PA 19107, USA

## Abstract

*Objective*. Stroke is a clinical diagnosis, with a history and physical examination significant for acute onset focal neurological symptoms and signs, often occurring in patients with known vascular risk factors and is frequently confirmed radiographically.* Case Report*. A 79-year-old right-handed woman, with a past medical history of hypertension, hyperlipidemia, and prior transient ischemic attack (TIA), presented with acute onset global aphasia and right hemiparesis, in the absence of fever or prodrome. This was initially diagnosed as a proximal left middle cerebral artery (MCA) stroke. However, CT perfusion failed to show evidence of reduced blood volume, and CT angiogram did not show evidence of a proximal vessel occlusion. Furthermore, MRI brain did not demonstrate any areas of restricted diffusion. EEG demonstrated left temporal periodic lateralized epileptiform discharges (PLEDs). The patient was empirically loaded with a bolus valproic acid and started on acyclovir, both intravenously. CSF examination demonstrated a pleocytosis and PCR confirmed the diagnosis of herpes simplex viral encephalitis (HSVE).* Conclusions*. HSVE classically presents in a nonspecific fashion with fever, headache, and altered mental status. However, acute focal neurological signs, mimicking stroke, are possible. A high degree of suspicion is required to institute appropriate therapy and decrease morbidity and mortality associated with HSVE.

## 1. Introduction

On average, someone in the United States has a stroke every 40 seconds [1], making stroke the fifth leading cause of death, and a major cause of adult disability in the United States according to the CDC. Prompt recognition, based primarily on history and physical exam, is paramount for timely diagnosis and treatment.

Herpes simplex viral encephalitis (HSVE) is also a neurological emergency. Timely recognition and treatment can alter the natural disease course from a mortality of approximately 70%, prior to the institution of acyclovir, to approximately 20–30% [2, 3]. HSVE often presents with nonspecific signs of encephalitis, including fever, headache, and altered mental status. Nevertheless, we present a case of acute herpes encephalitis presenting as a classic acute left MCA stroke, with global aphasia, left gaze deviation, and right sided hemiparesis, in the absence of fever, which delayed the diagnosis. Negative imaging, however, along with a high degree of suspicion, ultimately led to timely treatment and a good outcome.

## 2. Case

A 79-year-old right-handed woman, with a history of hypertension, hyperlipidemia, and prior TIA, currently prescribed clopidogrel, was taken to an outside hospital with acute onset aphasia and right sided weakness. The time she was last known well was at 21:30 h. Her NIHSS score was documented as 10 at 23:47, within the three-hour intravenous TPA window, and her initial computed tomography (CT) head was negative for intracranial haemorrhage. However, her systolic blood pressure was documented as 195–217 mmHg, and she received several boluses of intravenous labetalol, totaling 40 mg, without a decrease below the 185 mmHg threshold to receive TPA. TPA was withheld, and she was transferred to our institution for possible endovascular intervention.

On arrival, at 01:30 h the following morning and now 4 h from symptom onset, her blood pressure was 137/79 and her heart rate was 91 beats per minute and regular. Her temperature was 97.1°F (36.2°C). Her neurological exam was significant for global aphasia, left sided gaze deviation, right sided face, and arm and leg weakness. Her NIHSS score had increased to 15, with the following point distribution: level of consciousness (0), orientation (2), commands (2), gaze (1), visual fields (0), facial symmetry (1), left arm motor (0), right arm motor (2), left leg motor (0), right leg motor (3), ataxia (0), sensory (0), language (2), dysarthria (2), and extinction (0).

**Figure 1:**
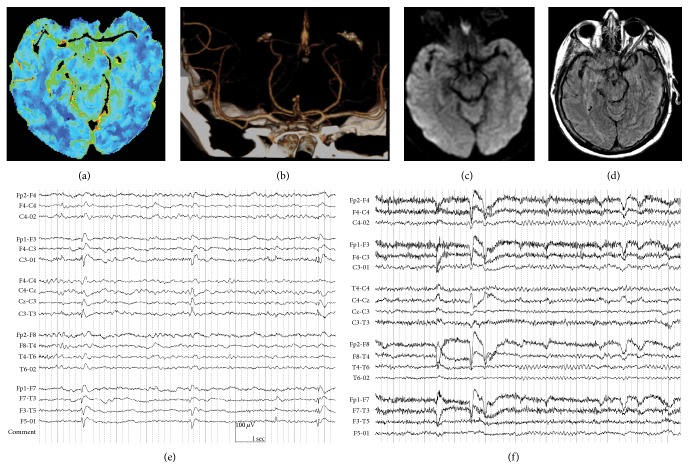
(a) Symmetric blood volume demonstrated on CT perfusion scan, in a 79-year-old woman presenting with a classic left MCA syndrome. (b) The absence of a proximal vessel cut-off in a CT angiogram reconstruction of the circle of Willis. (c) The absence of restricted diffusion in an MRI brain. (d) The absence of FLAIR hyperintensities indicative of an acute ischemic stroke or herpes simplex encephalitis. (e) Left anterior and midtemporal periodic lateralized discharges seen on hospital day 2, with a notable background which is diffusely slow, and an absence of a posterior dominant rhythm (PDR). (f) Return of the PDR of 8-9 Hz and the absence of PLEDs after the institution of intravenous valproic acid and acyclovir therapy.

A stat CT perfusion and CT angiogram were obtained (Figures 1(a) and 1(b)). There was no evidence of decreased blood flow or volume, and no evidence of a proximal vessel occlusion, which was unexpected given the density of her neurological findings on clinical exam. She was admitted to the acute stroke intermediate intensive care unit. Additionally, she was started on 3% hypertonic saline at 30 cc/h empirically in anticipation of cerebral edema, given the significance of her physical findings. She later developed a fever, with a temperature of 101.4°F (38.6°C), now approximately 11 h after symptom onset. A chest X-ray demonstrated bibasilar atelectasis (not shown), and admission lower extremity Doppler ultrasound showed no evidence of thrombus. Urine analysis demonstrated pyuria with moderate bacteria, and admission examination of her peripheral blood count demonstrated leukocytes totaling 10.2 × 10^3^/*μ*L. Two sets of blood cultures were obtained and she was started on broad spectrum antibiotics. Her temperature peaked on hospital day 2 at 103.3°F (39.6°C), now 22.5 h after symptom onset.

An MRI brain was obtained on hospital day 2, which demonstrated no evidence of restricted diffusion suggestive of a stroke (Figure 1(c)). Additionally, FLAIR imaging, although slightly motion degraded, showed only nonspecific white matter hyperintensities in the periventricular white matter, suggestive only of microangiopathy (Figure 1(d)).

The patient continued to demonstrate aphasia and right hemiparesis on hospital day 2. She was immediately loaded with intravenous valproic acid at 20 mg/kg and continued at 250 mg every eight hours thereafter. Additionally, the patient was empirically started on intravenous acyclovir at 10 mg/kg every eight hours. Furthermore, her antibiotics were broadened to include CNS penetrating fourth-generation cephalosporin. A stat EEG was ordered.

The family gave additional history of several prior lumbar spine injections for back pain, with the most recent approximately two months prior. As a result, empiric antifungal coverage was added with amphotericin B.

CSF examination on hospital day 2 demonstrated clear, colorless CSF, with an opening pressure of 29 cm H2O, CSF protein of 61 mg/dL (normal 15–55), CSF glucose of 89 mg/dL, and a CSF pleocytosis (in cells/*μ*L) consisting of 84 RBC and 7 WBC in tube 1 and 39 RBC and 11 WBC in tube 2, with a differential of 57% neutrophils and 19% lymphocytes in tube 4.

EEG on hospital day 2 demonstrated focal slow waves and focal sharps, which were periodic at times in the left frontotemporal and midtemporal regions, and slowing of the alpha rhythm (Figure 1(e)). These abnormalities normalized by hospital day 3, with a reemergence of a posterior dominant rhythm of 8-9 Hz (Figure 1(e)).

Additionally, the patient's physical exam began to improve; by hospital day 3, she was to move the right upper extremity against gravity. By hospital day 6, she was alert and oriented to person, place, and time, with 4/5 strength in the right upper extremity.

On hospital day 6, the CSF PCR returned positive for HSV type 1. On hospital day 7, the patient was transferred to an acute inpatient rehabilitation facility. She was to complete a 21-day course of acyclovir via peripherally inserted central catheter, and she was instructed to follow up in four weeks. However, she failed to return to the clinic for her follow-up appointment.

## 3. Discussion

HSVE has an annual incidence between 1 in 250,000 and 1 in 500,000 individuals per year, with HSV-1 being the most frequent cause of sporadic necrotizing encephalitis [4]. The clinical picture is often one of spontaneous and rapid developments of fever, headache, confusion, and seizures. Fever and abnormal mental status are the primary signs and symptoms of HSVE, occurring in >90% of patients. Approximately 50–60% of patients will present with nausea, vomiting, and meningismus. Seizures occur in about 50% of patients and focal neurological deficits appear in 30–50% [5]. Aphasia is also a common presentation, occurring in anywhere from one-third [6] to approximately 65% of HSVE cases found retrospectively [7, 8], leaving open the possibility for the misdiagnosis of HSVE as a stroke.

Several reports in the literature reinforce this. Townend and colleagues describe a case of an 84-year-old woman with confusion and reduced mobility, found to have HSV-1 encephalitis [9]. Differential diagnoses included delirium, sepsis, and possibly stroke, although their patient was febrile on presentation and had a prodromal, three-day history of symptoms. Additionally, Vachalová et al. describe a case of a 51-year-old woman with acute onset aphasia, after being admitted to hospital with fever and confusion [10]. HSVE can also present with ptosis, ophthalmoplegia, and contralateral hemiparesis, mimicking a Weber syndrome, despite, again, being preceded by a prodrome of fever, headache, and seizures [11]. AbdulJabbar and colleagues describe acute onset hemiparesis in a 17-year-old girl which preceded fever, altered mental status, and seizures by one week due to HSVE [12]. Still others have described a case of fever, headaches, and altered mental status for approximately 1 week, misdiagnosed as an ischemic stroke based on right sided frontal and temporal hypodensities noted radiologically and later found to have HSVE [13].

Several reports of aphasia or weakness attributed to HSVE exist; however, the complete syndrome of acute onset global aphasia, left gaze deviation, and right sided hemiparesis, in the absence of fever, has not, to our knowledge, been previously described. Furthermore, the classically described temporal lobe hyperintensities were notably absent, leaving the diagnosis based on strong clinical suspicion, confirmed by EEG and PCR of HSV DNA from the CSF.

While the potential for HSV to mimic stroke is present, there are only a few case reports in the literature. In contrast, a recent study from the UK on 1165 patients admitted to an acute stroke unit reported only 163 (14%) cases were medical mimics, and of those, none were classified as encephalitis [14], suggesting it is not frequently encountered among the more common stroke mimics.

Often, both MRI and EEG are able to aid in the diagnosis of HSVE. On MRI, lesions due to HSVE are characterized by restricted diffusion, which results from cytotoxic edema. This can potentially be mistaken for stroke, especially when involving only one vascular distribution [15]. HSV has a predilection for the temporal lobes and often can be seen as increased T2/FLAIR signal intensity, which is highly specific, but not sensitive [6, 16]. In a retrospective analysis of all encephalitides with temporal lobe lesions on MRI, HSVE was most common and less likely to present with involvement outside of the temporal lobe, insula, or cingulate or show involvement of the bilateral temporal lobes [6].

Given the hemorrhagic nature of HSVE, it is uncharacteristic that the MRI was negative for any signal abnormalities, given the patient's neurological exam. It is feasible that a follow-up MRI may have demonstrated the expected T2/flair and restricted diffusion signal abnormalities that are commonly associated with HSVE. However, because the patient had improved considerably on our therapy, and because PCR analysis demonstrated the HSV-1 organism, a second follow-up MRI was not pursued.

As was demonstrated by our case, EEG was able to localize lesions in 81% of HSVE cases as compared to 50%–59% by CT [17]. Findings by EEG indicative of HSVE were predominantly periodic discharges and focal slowing, localized to the area of the brain involved, and found in 65% of proven cases [17, 18]. Also, these EEG patterns occur in significantly greater proportions in HSVE, as compared to other non-HSV and autoimmune encephalitis [18]. Furthermore, a normal EEG was an independent predictor of a good outcome [18], whereas 93% of patients with HSVE had abnormal EEGs in a separate retrospective study [19].

Ultimately, diagnosis via PCR of the double stranded HSV genome is currently the accepted gold standard. It has both a high degree of sensitivity (98%) and specificity (94%) for diagnosis of the disease and, in most cases, obviates the need for brain biopsy to establish a diagnosis [16, 20, 21].

Untreated, the mortality of HSVE approaches 70%. Intravenous acyclovir decreases the mortality to approximately 30%; however, survivors may have permanent neurological deficits [2, 3, 19]. Normal function was reestablished in 38% of patients who received acyclovir. Patients with a Glasgow coma score of <6, those aged >30 years, and those with encephalitis of >4 days' duration were significantly more likely to have a poor outcome [20]. In a separate study, a delay of greater than 2 days in hospital before the institution of intravenous acyclovir therapy was independently associated with poor outcome, that is, severe disability or death at 6 months [22]. Often, the most common reason for the delay was a failure to consider HSVE among the initial diagnostic possibilities [23].

Because intravenous TPA is a highly time sensitive intervention, ischemic stroke is often the first diagnosis to be ruled out when a patient presents with acute onset focal deficits, especially in the absence of fever or prodrome. Many institutions obtain immediate CT angiogram and CT perfusion along with a noncontrast CT head in the triage of acute stroke patients. Often the MRI, which is more sensitive for stroke, is obtained later in the hospitalization. However, in a pooled meta-analysis, CT perfusion had a sensitivity of 80% and a specificity of 95%, with the majority of false negatives attributed to small lacunar infarcts [24]. Considering a diagnosis of HSVE with a negative CT perfusion study, before the MRI was obtained, may have decreased the delay in diagnosis of HSVE by almost one day.

It is possible for HSVE to present with acute onset neurological deficits, in the absence of prodrome or seizures. A high index of suspicion is required to initiate timely therapy and to maximize the probability of a good prognosis.
